# Surgical Treatment of Otosclerosis: Eight years’ Experience at the Jordan University Hospital 

**Published:** 2013-10

**Authors:** Tareq Mahafza, Abdelmonem AL-Layla, Mohammed Tawalbeh, Yagoub Abu-yagoub, Ahmad Atwan Sulaiman

**Affiliations:** 1 *Department of *Otorhinolaryngology,* University of Jordan, Amman- Jordan.*; 2 *Department of Public health, University of Jordan, Amman- Jordan.*

**Keywords:** Jordan University Hospital, Otosclerosis, Surgical treatment

## Abstract

**Introduction:**

To report the experience of the Jordan University Hospital with respect to the surgical treatment of otosclerosis and to compare results and complications with published studies.

**Materials and Methods:**

The medical records of all patients who underwent stapes surgery for otosclerosis at the Jordan University Hospital during the period January 2003 to December 2010 were reviewed.

**Results:**

Out of 130 patients who underwent stapes surgery, 104 (80%) fulfilled the criteria and were enrolled in this study. There were 68 (65.4%) females and 36 (34.6%) males (female-to-male ratio, 1.9: 1). The disease was bilateral in 86 (82.7%) patients. Family history for otosclerosis was positive in 37(35.6%) patients. Tinnitus was observed at presentation in 82 (78.8%) patients and spontaneously resolved or improved in 51(62.2%) patients after surgery. Air bone gap after surgery was ≤ 10 dB in 79 (76.0%) patients, between 10 and 20 dB in 10 (9.6%) patients, and between 20 and 30 dB in four (3.8%) patients. Complications occurred in 17(16.3%) patients, which included: one (1.0%) deaf ear, two (2.0%) sensorineural hearing loss, two (2.0%) facial nerve palsy, six (5.8%) vertigo lasting more than 7 days, three (2.9%) chorda tympani damage, one (1.0%) floating of footplate, and two (2.0%) perforation of the tympanic membrane.

**Conclusion:**

The results of this study are comparable with those reported in the literature by surgeons with the same level of experience, but below than those with large series experience. Therefore, we believe that an experienced general ear, nose, and throat (ENT) surgeon can perform stapes surgery safely and successfully in the absence of an otologist.

## Introduction

Otosclerosis is a disease of the otic capsule and middle ear ossicles, in which a new dense sclerotic bone is formed (1). The etiology of otosclerosis has not been fully elucidated despite numerous studies; however many theories have been suggested to explain it on the basis of genetic, viral, hormonal, and other factors (2-5). The inheritance of otosclerosis is believed to be predominantly autosomal with variable penetrance, but other modes of inheritance are possible (6). Caucasians are more commonly affected by otosclerosis than Asian, black, and Native American populations, with a prevalence of clinical otosclerosis of less than 1% among white individuals (7-9). Females are disproportionately affected in a variable ratio to males (7,10-13). Otosclerosis is a bilateral disease in approximately 80% of cases (14). Patients present with gradual conductive hearing loss and with tinnitus in approximately 65–92% of cases (15,16).

Treatment options for otosclerosis include medications, use of hearing aids, and surgery. Medical treatment is indicated in the early active stage of the disease, which usually goes unnoticed, while hearing aids tend to be indicated when patients refuse surgery. The surgical treatment of otosclerosis is the most commonly used and most effective treatment and includes either stapedectomy or stapedotomy. Shea first performed stapedec- tomy in 1956 and is considered the pioneer of the modern surgical treatment of otosclerosis (17). Stapedotomy, which is an opening into the footplate of the stapes, or stapedectomy, which is a total removal of the stapes, are both successful in the treatment of otosclerosis, but most otologists prefer stapedotomy since it has fewer complications than stapedectomy (18,19).

In stapes surgery, the success rate for achieving a postoperative air bone gap (ABG) of less than 10 dB is close to 95% in large series (20-22), while in studies with a smaller series this rate decreases to a mean of 80% (23-26). Variable complications may occur after stapes surgery, but the most worrisome complication is sensorineural hearing loss (SNHL) which occurs in less than 0.5% of patients undergoing surgery by experienced surgeons who have a large series (20-22).

The aim of this study was to review the experience with the surgical treatment of otosclerosis at the Jordan University Hospital and to compare it with other reported outcomes.

## Materials and Methods

A retrospective review of records of all patients who underwent stapes surgery for otosclerosis at the Jordan University Hospital from January 2003 to December 2010 was conducted. The data collected included: age, gender, ear involved, presence of tinnitus, surgical technique used, abnormalities found in the ear, results of surgery, and presence of complications. Patients who had revision surgery, patients lost to follow-up, and patients with incomplete data were all excluded.

All patients underwent pure tone audiogram (PTA) in a standard soundproof room including air conduction (AC), bone conduction (BC) before and after the stapes surgery, and ABG calculated at the standard frequencies of 0.5,1,2,4 kHz as recommended by the committee on hearing and equilibrium at the American Academy of Otology (27).

Surgery was performed under general anesthesia using the same standard surgical technique with a micro-drill or perforator. The oval window was sealed with a fascia or perichonderium and a Teflon piston prosthesis was used in the majority of cases. The follow-up policy involved examination of patients at intervals of 1 week, 1,3,6 months and then yearly after surgery and at each visit for pure tone audiography. Hearing loss was classified according to the limits defined by the American Medical Association: mild hearing loss, between 20 and 40 dB; moderate, 40–55 dB; moderately severe, 55–70 dB; severe, 70–90 dB; profound, >90 dB.

Hearing results were calculated from the last audiogram performed preoperatively and an audiogram performed 1 year after stapes surgery. ABG was calculated by subtracting the preoperative BC thresholds from the postoperative AC thresholds, and surgery was considered successful if the ABG was less than 10 dB, and satisfactory if the ABG was between 11 and 30 dB, while any improvement in which the ABG was greater than 30 dB was considered unsatisfactory. If the ABG was the same or worse the results were considered as a failure. Furthermore, the improvement in air and BC thresholds were calculated by subtraction of postoperative from preoperative values. In all records there was a routine question to patients about their satisfaction with surgery with respect to hearing and tinnitus, which was answered by all patients. 

Data collected were analyzed using the student t-test for paired samples and differences were considered statistically significant if the P value was ≤ 0.05.

## Results

Out of 130 patients who underwent stapes surgery, 104 (80.0%) fulfilled the criteria and were enrolled in this study. As shown in Table 1, there were 68 (65.4%) females and 36 (34.6%) males, with a ratio of 1.9: 1.

The mean age at presentation was 43 years (SD = 11.12) and the average duration of the disease was 6.2 years. The disease was bilateral in 86 (82.7%) patients and unilateral in 18 (17.3%) patients, with a right-to-left ear ratio of 1.17: 1. The family history for otosclerosis was positive in 37 (35.6%) patients, and the degree of hearing loss at presentation was moderate or moderately severe in more than 80% of patients. Tinnitus was observed at presentation in 82(78.8%) patients, and resolved after stapes surgery in 22(26.8%) patients and improved in 29 (35.4%) patients with an overall 62% success rate.

**Table 1:** Demographic Data of 104 patients

	**Number ** **(%)**
Mean age at presentation in years Age range in years	43 17–68
SEX Females Males Female: male ratio 1.9: 1	68 (65.4) 36 (34.6)
Preoperative tinnitus	82 (78.84)
Family history of Otosclerosis	37 (35.57)
Bilateral Otosclerosis Right ear involved Left ear involved Left to Right Ratio	86 (82.69) 48 (46.1) 56 (53.9) 1.17 : 1

The hearing results obtained are depicted in Tables 2 and 3. As shown in Table 2, the ABG was ≤ 10 dB in 79 (76.0%) patients, between 10 and 20 dB in 10 (9.6%) patients, between 20 dB and 30 dB in four (3.8%) patients, and greater than 30 dB in eight (7.69%) patients. Three (2.9%) patients developed SNHL. 

**Table 2:** Hearing results with regards to ABG*

**ABG/ dB**	**Number (%)**
≤ 10	79 (75.96)
<10≤ 20	10 (9.61)
<20≤ 30	4 (3.84)
<30	8 (7.69)
No ABG, But SNHL**	3 (2.88)

*ABG: Air bone gap **SNHL: Sensorineural hearing loss

In Table 3, the mean value of AC threshold for frequencies 0.5–4 kHz was 49.8 dB before surgery and 25.6 dB after surgery, with a statistically significant mean difference (hearing improvement) of 24.2 dB (P≤ 0.05). The mean value of the BC threshold for the same 0.5–4 kHz frequencies was 23.6 before surgery and 20.5 dB after surgery, with a statistically significant mean difference (hearing improvement) of 3.1dB (P≤0.05). In response to the question about patients’ satisfaction with a stapes surgery, 80 (76.9%) patients reported satisfaction and 24 (23.1%) were unsatisfied with their stapes surgery. Complications that occurred either during or after stapes surgery were detected in 17 (16.3%) patients and are summarized in Table 4. Complications included one (1.0%) case of deaf ear, two (2.0%) cases with SNHL, two (2.0%) cases with temporary facial nerve palsy (grade 2), six (5.8 %) cases with vertigo lasting more than 7 days, three (2.9%) cases with chorda tympani damage, one (1.0%) case with floating of footplate, and two (2.0%) cases with perforation of the tympanic membrane which was dealt with by grafting during surgery. Two cases who developed SNHL and four cases who developed vertigo underwent total stampede- ctomy without sealing of the oval window, because there was a narrow oval window niche.

**Table 3:** Mean of hearing threshold before and after stapes surgery

	**Preoperative**	**Postoperative**	**Mean Gain**
Mean Air Conduction Threshold (dB)*	49.6	25.6	24.2
Mean Bone Conduction Threshold (dB)	23.6	20.5	3.1

*dB- deci Bell

**Table 4:** Complications of stapes surgery

**Complications**	**Number (%)**
SNHL	3 (2.88)
Facial palsy	2 (1.92)
Vertigo lasting < 7 days	6 (5.76)
Chorda tympani injury	3 (2.88)
Floating footplat	1(.96)
Perforation	2 (1.92)
Total	17 (16.34)

## Discussion

Otosclerosis is an inherited disease with autosomal dominant mode of inheritance in 40–54% of cases (28). In this study, 37 (35.57%) of cases had a positive family history of otosclerosis.

Females suffer from otosclerosis more commonly than males, with a variable female-to-male ratio of up to 3:1 (29). Among patients in this study there were 68 (65.4%) females and 36 (34.6%) males, with a female-to-male ratio of 1.9:1, which is consistent with the average of female-to-male ratio (2:1) (24,29). Bilateral involvement of the ears with otosclerosis in this study was noted in 86 (82.7%) patients, which is within the range of the international figures (14). The mean age of patients in this study at presentation, and consequently at the time they had the stapes surgery, was 43 years, which is consistent with most other reports (40–50 years) (19,30). 

Hearing loss is always a presenting symptom, but tinnitus presents in 65–92% cases (15-17). The rate in this study, 78.8%, was within the published range. After stapes surgery,22(26.8%) patients reported complete resolution and 29 (35.36%) patients reported improvement of the tinnitus, with an overall success rate of 62%. This is consistent with previously reported figures (3–78%) (31).

The majority of studies on the surgical treatment of otosclerosis have demonstrated good short- and long-term hearing results regardless of the surgical techniques used (18,19,32). However, the majority of otolaryngologists still prefer stapedotomy over stapedectomy because it has a lower rate of complications (32,33), and that is why this technique was adopted in the majority of patients involved in this study.

The stapes surgery success rate, in which ABG ≤ 10, among surgeons with a large series (20-22) is close to 95%; but in other studies with smaller series (23-26,34), the success rate decreases, but remains very good. Very good results (ABG ≤ 10 dB) were achieved in this study in 79 (76.0%) patients and satisfactory results (< 10 ABG ≤ 30 dB) in 14 (13.5%) patients, with an overall success of 89.4%. These results are considered very good and comparable with most studies with a limited number of cases (23-26,34). 

The other factor which is taken into consideration in evaluating the results of stapes surgery is the hearing gain in AC threshold. A 26.2-dB hearing gain was achieved in this study, and again this is comparable with previously reported results (26,32,35).

Intraoperative and postoperative compli- cations occurred in 17 (16.3%) of patients, but most of these were transient with the exception of SNHL and vertigo that lasted more than 7 days but did not cause any concern. The rate of perioperative and postoperative complications in this study were similar to previous international reports. One complication was a dead ear and two cases of SNHL, representing 2.92% of the operations performed. The rate of SNHL in this study is still within the average rate reported in the literature (0.4% to 3%) for primary stapes surgery (36).

Vertigo is a common complaint in the first few days after stapes surgery, but it rarely lasts more than a week and usually affected patients develop a permanent vestibular hypofunction after a while and become adapted to the condition (37). In 1985, Birch and Elbrond reported a rate of 4% of vertigo lasting more than a week, as compared with 5.8% in this study (37).

## Conclusions

The results of this study are very good and comparable with those reported in the international literature with a similar number of patients, although lower than those from surgeons who have large number of patients. Therefore we believe that an experienced general ENT surgeon can perform the stapes surgery safely and successfully in the absence of an experienced otologist who is likely to achieve better results.

## References

[1] Schuknecht HF (1974). Pathology of the ear.

[2] Moumoulidis I, Axon P, Baguley D, Reid E (2007). A review on the genetics of otosclerosis. Clin Otolaryngol.

[3] Arnold W, Friedmann I (1987). Detection of measles and rubella-specific antigens in the endochondral ossification zone in otosclerosis. Laryngol Rhinol Otol.

[4] Precechtel A (1967). Determination of the effect of pregnancy on activation of otosclerosis. Acta Otolaryngol.

[5] Yoo TJ (1984). Etiopathogenesis of otosclerosis: a hypothesis. Ann Otol Rhinol Laryngol.

[6] Sabitha R, Ramalingam R, Ramalingam KK, Sivakumaran TA, Ramesh A (1997). Genetics of otosclerosis. J Laryngol Otol.

[7] Altman F, Glasgold A, Macduff JP (1967). The incidence of otosclerosis as related to race and sex. Ann Otol Rhinol Laryngol.

[8] Karosi T, Sziklai I (2010). Etiopathogenesis of otosclerosis. Eur Arch Otorhinolaryngol.

[9] Cajade FJ, Labellero T (2003). Epidemiological aspects of Otosclerosis (1). Its frequency in comparison with other ear pathologies, incidence and prevalence. Ann Otorhinolaryngol Am.

[10] Arnold W, Busch R, Arnold A, Ritsher B, Neiss A (2007). The influence of measles vaccination on the incidence of otosclerosis in Germany. Eur Arch Otorhinolaryngol.

[11] Redfors YD, Möller C (2011). Otosclerosis: Thirty-year’s follow-up after surgery. Ann Otol Rhinol Laryngol.

[12] House JW, Cunningham CD 111. Otosclerosis, Flint PW, Haughey BH, Niparko JK (2010). Cummings CW. Cummings Otolaryngology–Head and Neck Surgery.

[13] Nemati S, NaghaviI E, Kaemnejad E (2013). Middle ear exploration results in suspected otosclerosis cases: Are ossicular and footplate area anomalies rare. Iran J Otorhinolaryngol.

[14] Hueb MM, Goycoolea MV, Paparella MM, Oliveira JA (1991). Otosclerosis: The University of Minnesota temporal bone collection. Otolaryngol Head Neck Surg.

[15] Oliveira CA (2007). How does stapes surgery influence severe disabling tinnitus in otosclerosis patients. Adv Otorhinolaryngol.

[16] Gristwood RE, Venables WN (2003). Otosclerosis and chronic tinnitus. Ann Otol Rhinol Laryngol.

[17] Shea JJ (1956). Symposium on the operation for mobilization of the stapes in otosclerosis. Laryngoscope.

[18] House HP, Hansen MR, Al Dakhail AAA, House JW (2002). Stapedectomy versus stapedotomy: comparison of results with long term follow-up. Laryngoscope.

[19] Aarnisalo AA, Vasama JP, Hospu E, Ramsay H (2003). Long term hearing results after stapes surgery. A 20 year follow-up. Otol Neurotol.

[20] Shea JJ Jr (1998). Forty years of stapes surgery. Am Otol.

[21] Vincent R, Sperling NM, Oates J, Jindal M (2006). Surgical findings and long term hearing results in 3050 stapedectomies for primary otosclerosis: a prospective study with the otology-neurotology database. Otol Neurotol.

[22] Lippy WH, Battista RA, Bernholz I, Schuring AG, Burkey JM (2003). Twenty year review of revision stapedectomy. Otol Neurotol.

[23] Constantinidis I, Vaz F, Triaridis S, Fairley JW (2002). Causse laser stapedectomy. Results and patient’s satisfaction rate audit in a district general hospital. Hippokratia.

[24] Ramsay H, Kärkkäinen J, Palva T (1997). Success in surgery for otosclerosis: hearing improvement and other indicators. Am J Otolaryngol.

[25] Salahuddin I, Salahuddin A (2002). Experience with stapedectomy in a developing country: A review of 200 cases. Ear Nose Throat J.

[26] Simoncelli C, Ricci G, Trabalzini F, Gullà M, Faralli M, Molini E (2005). Stapes surgery: A review of 515 operations performed from 1988 to 2002. Mediterr J Otol.

[27] Monsell EM, Balkany TA, Gates GA, Goldenberg RA, Meyerhoff WI, House JW (1995). Committee on Hearing and Equilibrium Guidelines for Evaluation of Results of Treatment of Conductive Hearing Loss. Otolaryngol Head Neck Surg.

[28] Cawthorne T (1955). Otosclerosis. J Laryngol Otol.

[29] Emmett JR (1993). Physical examination and clinical evaluation of patients with otosclerosis. Otolaryngol Clin North Am.

[30] Vartiainen E (1999). Sex differences in patients with hearing impairments caused by otosclerosis. Eur Arch Otorhinolaryngol.

[31] Causse J, Vincent R (1995). Poor vibration of inner ear fluids as a cause of low tone tinnitus. Am J Otol.

[32] Fisch U (1982). Stapedectomy Versus stapedotomy. Am J Otol.

[33] Shea JJ (1982). Stapedectomy-long- term report. Ann Otol Rhinol Otol.

[34] Chin-Lung K, Mao-Che W, Chia-Huei C (2013). The influence of crimping of nitinol and conventional prostheses on hearing success for otosclerosis. Int Adv Otol.

[35] Ueda H, Miyazawa T, Asahi K (1999). Factors affecting hearing results after stapes surgery. J Laryngol Otol.

[36] Glasscock ME, 3rd, Storper IS, Haynes DS (1995). Twenty five years of experience with stapedectomy. Laryngoscope.

[37] Birch L, Elbrond O (1985). Stapedectomy and vertigo. Clin Otolaryngol.

